# Hippocampal place codes are gated by behavioral engagement

**DOI:** 10.1038/s41593-022-01050-4

**Published:** 2022-04-21

**Authors:** Noah L. Pettit, Xintong C. Yuan, Christopher D. Harvey

**Affiliations:** Department of Neurobiology, Harvard Medical School, Boston, MA USA

**Keywords:** Navigation, Hippocampus

## Abstract

As animals explore an environment, the hippocampus is thought to automatically form and maintain a place code by combining sensory and self-motion signals. Instead, we observed an extensive degradation of the place code when mice voluntarily disengaged from a virtual navigation task, remarkably even as they continued to traverse the identical environment. Internal states, therefore, can strongly gate spatial maps and reorganize hippocampal activity even without sensory and self-motion changes.

## Abstract (web summary)

The authors found that the expression of spatial maps in the hippocampus is modulated by the internal state of an animal. Thus, the brain’s code for spatial positions within an environment can transform even without changes to the external world.

## Main

The hippocampus forms spatial codes that are critical for navigation^1–4^. These codes are based on features of the environment, including sensory cues and the location of rewards^5–8^. Indeed, hippocampal codes remap when animals are exposed to environments with distinct sensory cues or different task requirements and when cues and rewards are moved within an environment^9,10^. In addition, spatial codes are based on egocentric information, including self-motion^11,12^, and often degrade when animals are not actively moving themselves through an environment^13–15^.

Models of the hippocampus propose that sensory and self-motion signals combine to automatically form a spatial code in a self-supervised manner when an animal actively moves through an environment and experiences sensory cues^12,15–19^. Consistent with this idea, place codes form rapidly in new environments even before animals understand the behavioral relevance, such as before experiencing rewards^20,21^. Also, place codes are present even during behaviors that likely do not require hippocampal activity or detailed spatial maps, such as random foraging^22,23^. Moreover, place cells are easily identified across a wide range of experimental settings. Together, these results suggest that the hippocampus always maintains a spatial map of an environment during movement, and this spatial code is remapped selectively when the environment changes.

**Fig. 1:**
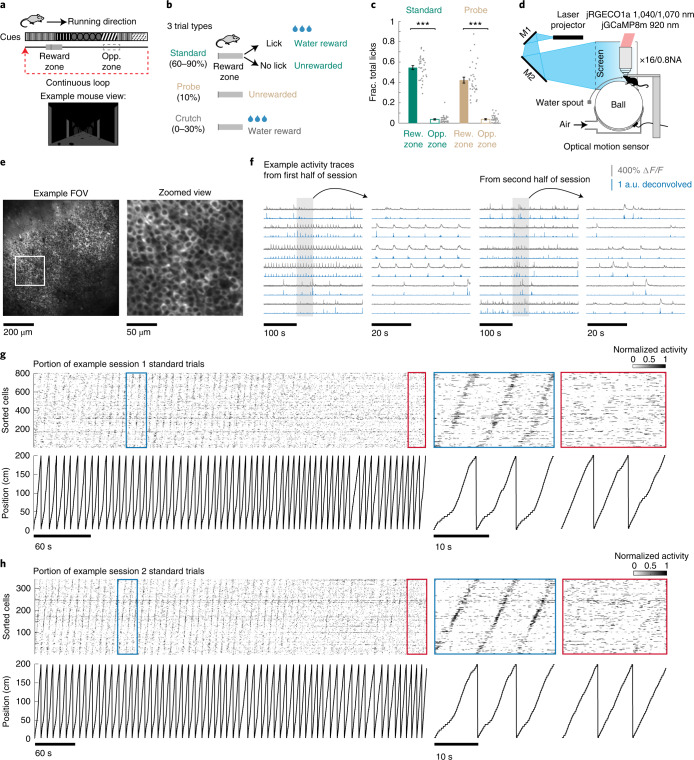
The hippocampal spatial code changed despite continuous traversal of the same environment. **a**, Mice traversed a 2-m-long virtual linear track with visual cues that repeated in a circular topology. The start of the true reward zone is indicated by a gray vertical line; note that all analyses consider a peri-reward region, including the 10 cm immediately before the true reward zone. For simplicity, we refer to this peri-reward region as the reward zone. **b**, Three trial types were interleaved. Licking in the true reward zone on standard trials triggered water delivery. Probe trials omitted rewards. Crutch trials delivered rewards regardless of licking. **c**, Trained mice showed preferential licking in the reward zone on probe and standard trials. Consumption licks were excluded. Error bars: mean ± s.e.m. Raw data points jittered horizontally are shown next to each bar. Two-sided Wilcoxon signed-rank test: reward versus opposite zones on standard trials, *P* = 8.0 × 10^−7^; reward versus opposite zones on probe trials, *P* = 8.0 × 10^−7^. *n* = 32 sessions and 8 mice. **d**, Schematic of behavioral and imaging setup. M1 and M2 are mirrors. **e**, Left: representative imaging field of view. Right: zoomed view. Similar fields of view were obtained from 39 sessions that met inclusion criteria. **f**, Example jGCaMP8m Δ*F*/*F* and deconvolved activity traces from the first and second half of a session. Six cells are shown. **g**, **h**, Portion of two example sessions (standard trials only). Top: raster plots of neural activity on each frame, sorted by the location of each neuron’s maximal activity on correct trials. Only neurons whose standard deviation of activity is above the 30th percentile of all neurons’ standard deviation are shown. All neurons’ activities were normalized so that the top 8% pixels of the raster are saturated. Bottom: corresponding linear track position. a.u., arbitrary unit; FOV, field of view.

In this study, we took advantage of voluntary changes in a mouse’s engagement in a goal-directed navigation task to test whether the conjunction of sensory and self-motion signals is sufficient for hippocampal spatial coding or if internal states modulate place codes. Mice were trained in virtual reality to navigate a 2-m-long linear track that repeated in a circular topology (Fig. 1a). Mice received liquid rewards if they licked a spout in a 20-cm-long reward zone, whereas licks in other parts of the track were unrewarded^23^ (Fig. 1a,b). Only one reward was available on each lap. In a subset of trials (‘crutch trials’), we gave a reward in the reward zone even without a lick to help mice learn the reward location (Fig. 1b). In other trials (‘probe trials’), we omitted the reward to test the mouse’s behavior regardless of consumption licks (Fig. 1b). Trained mice exhibited selective licking near the reward zone, including in probe trials, indicating a memory of the reward location (Fig. 1c).

**Extended Data Fig. 1:**
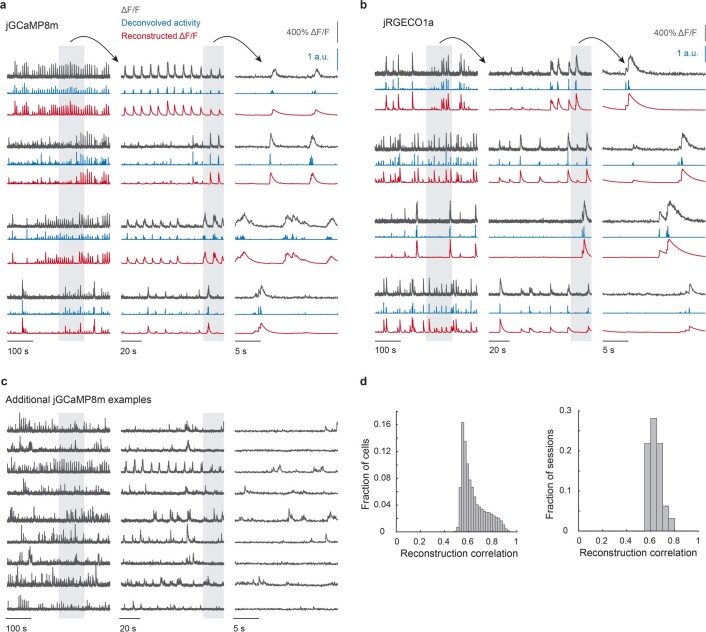
Example fluorescence traces and deconvolved activity. **a**. Representative jGCaMP8m ∆F/F, deconvolved activity, and reconstructed ∆F/F for four cells, at different temporal scales. Shaded regions in the columns 1 and 2 indicate expanded regions in columns 2 and 3, respectively. **b**. Representative jRGECO1a traces, plotted as in (a). **c**. Additional example jGCaMP8m ∆F/F traces. **d**. Histograms of correlation between ∆F/F and reconstructed ∆F/F from deconvolution across cells (left) and sessions (right).

**Extended Data Fig. 2:**
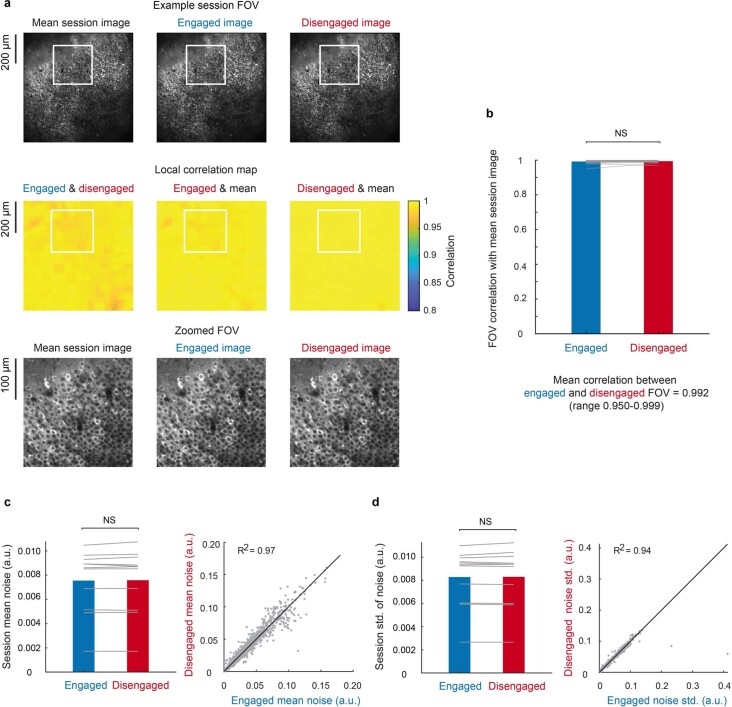
Stability of the imaging field of view. **a**. Example session mean image, image from the engaged trials, and image from the disengaged trials, and local correlation map between them. For each pixel *x* in the FOV, the local neighborhood used for correlation calculation is a square of 41 by 41 pixels with *x* in the center. Similar results were obtained from 32 sessions that met inclusion criteria and contained more than 10 engaged and disengaged trials. **b**. Correlation between the mean session FOV image and FOV images of the engaged and disengaged trials. Two-sided Wilcoxon signed rank test, p = 0.06. The mean correlation between FOVs in the engaged and disengaged trials is 0.992. n = 32 sessions, 8 mice. **c**. Mean noise in the engaged and disengaged trials. Noise was defined as the activity less than 2 standard deviations from the mean activity of each cell. Two-sided Wilcoxon signed rank test, p = 0.46. n = 32 sessions, 8 mice. **d**. Standard deviation of noise in the engaged and disengaged trials. Two-sided Wilcoxon signed rank test, p = 0.97. n = 32 sessions, 8 mice.

We measured the activity of hundreds of CA1 neurons using cellular-resolution calcium imaging^24^ (Fig. 1d–f and Extended Data Fig. 1). As expected, we observed place cell activity in a large fraction of neurons, forming a sequence that tiled the entire track (Fig. 1g,h). Surprisingly, however, toward the end of some sessions, this sequential pattern disappeared, and the same population of neurons shifted to distinct activity patterns (Fig. 1g,h). This change in activity occurred even though the mouse continued to traverse the track and ran past identical visual cues on all trials. Notably, our imaging field of view was stable throughout the session (Extended Data Fig. 2).

**Fig. 2:**
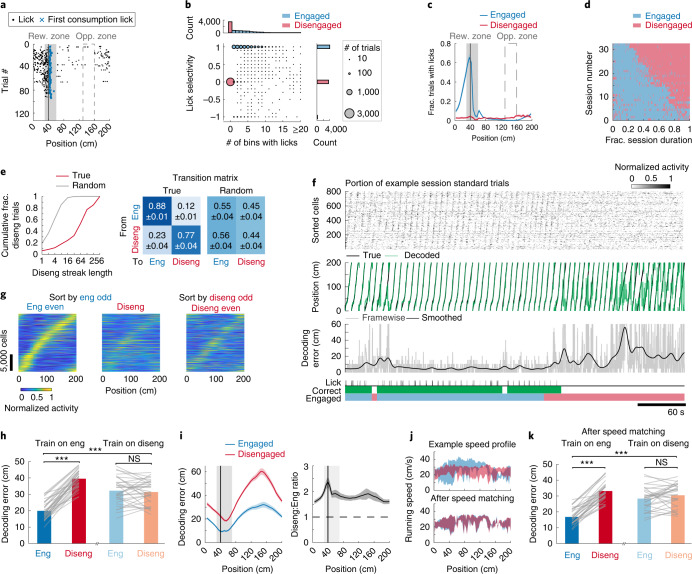
The hippocampal spatial code degraded as mice disengaged from the task. **a**, Lick raster of an example session with 125 trials. The reward zone (gray shaded area) includes anticipatory licking before reward availability (solid vertical line). The first consumption lick is indicated, whereas subsequent consumption licks inside the reward zone were excluded. **b**, The trial-wise lick selectivity and number of 5-cm bins with licks are shown for the 39 sessions that met the inclusion criteria. Circle sizes corresponds to the number of trials. The color code corresponds to k-means clustering with two clusters. The ‘engaged’ cluster had higher lick selectivity and number of bins with licks. **c**, Summary of lick behavior across 32 sessions that had more than ten disengaged trials. Shading represents mean ± s.e.m. **d**, Distribution of engaged and disengaged trials across 32 sessions. **e**, Left: distribution of disengaged trials in streaks of different length. Right: transition matrix between trial types averaged across sessions. Mean ± s.e.m. is noted. **f**, Portion of the standard trials from the same session as Fig. 1g. Decoded positions and decoding error were calculated using decoders trained on sliding windows of 20 trials and tested on the immediate next trial. Chance-level error is 50 cm. Licks, correctness and engagement status (blue: engaged; red: disengaged) are shown at the bottom. **g**, Sequence plots of cells with significant place fields in correct standard trials pooled across sessions. Cells were sorted by the location of peak activity using the trials indicated. Each cell’s activity was percentile normalized, saturating the top and bottom 2%. **h**, Decoding performance on held-out trials. The decoder was trained using ten trials of one engagement type at a time and tested on all other trials. The mean trial-wise decoding error was calculated from all iterations of the decoder. Each gray line indicates one session. Chance-level error is 50 cm. Two-sided Wilcoxon signed-rank test: engaged versus disengaged for decoder trained on engaged trials, *P* = 8.0 × 10^−7^; engaged versus disengaged for decoder trained on disengaged trials, *P* = 0.78. Train/test on engaged versus train/test on disengaged, *P* = 2.2 × 10^−6^. *n* = 32 sessions and 8 mice. **i**, Left: mean framewise decoding error by position for the decoder trained on engaged trials. Right: ratio of disengaged to engaged decoding error by position. Shading represents mean ± s.e.m. *n* = 32 sessions and 8 mice. **j**, Top: example speed profile (interquartile range) of ten engaged and ten disengaged trials. Bottom: speed profile of the same trials after speed matching. **k**, Same as **h** but using speed-matched frames for decoding. Two-sided Wilcoxon signed-rank test: engaged versus disengaged for decoder trained on engaged trials, *P* = 1.7 × 10^−6^; engaged versus disengaged for decoder trained on disengaged trials, *P* = 0.06. Train/test on engaged versus train/test on disengaged, *P* = 2.9 × 10^−6^. diseng, disengaged; eng, engaged; NS, not significant.

The shift in neural activity patterns appeared to coincide with voluntary disengagement of the mouse from the task, which was apparent as a reduction of licking and a decline in the fraction of trials that the mouse performed correctly (Fig. 2a). We developed two lick-based metrics to characterize the change in task engagement in well-trained mice. Lick selectivity quantifies the level of licking in the peri-reward zone compared to an equally sized zone on the opposite side of the track. We also calculated the number of spatial bins with licks on each trial as a measure of the amount of licking throughout the track. An engaged mouse is expected to lick during the trial to trigger a reward and show preferential licking at the reward zone, whereas a disengaged mouse may lick less and without spatial selectivity. We calculated these two metrics on individual trials and clustered the trials into two groups. One cluster had high lick rate and selectivity, which we termed ‘engaged’ trials, and the other had low lick rate and selectivity, which we termed ‘disengaged’ trials (Fig. 2b,c).

**Extended Data Fig. 3:**
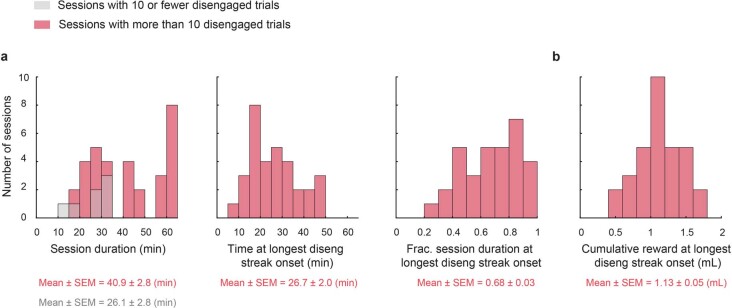
Session duration and reward volume at the onset of the longest disengaged streak. **a**. Histograms of session duration, onset time of the longest disengaged streak, and the fraction of total session duration at the onset of the longest disengaged streak. **b**. Histograms of cumulative reward volume at the longest disengaged streak onset.

Some sessions had nearly exclusively engaged trials, whereas others had appreciable numbers of both and were the focus of this study. Disengaged trials typically occurred at the end of the session when mice had received close to 1 ml of rewards and occurred in streaks that were longer than expected by chance (Fig. 2d,e and Extended Data Fig. 3). As a result, there was a high probability that a disengaged trial was followed by another disengaged trial, and the same was true for engaged trials, indicating that sessions had separate periods of engagement and disengagement (Fig. 2e). Thus, mice appeared to switch from an engaged to disengaged behavioral state, often near the end of a session.

**Fig. 3:**
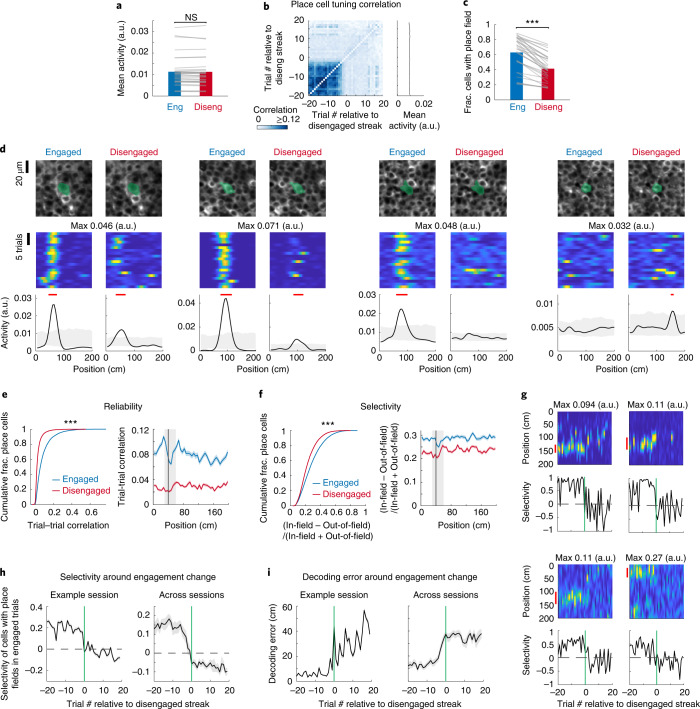
The disengaged trials had fewer place cells and lower quality place fields. **a**, Mean population activity in the engaged and disengaged trials. Each gray line indicates one session. *n* = 32 sessions and 8 mice. Two-sided Wilcoxon signed-rank test, *P* = 0.70. **b**, Left: trial–trial correlation of spatially binned activity averaged across cells of all sessions on 20 trials (at least 80% engaged) immediately before a streak of at least ten disengaged trials. *n* = 12 sessions and 6 mice. Values along the diagonal were set to 0. The top 1% of correlation values were saturated. Right: mean trial-wise activity of all cells on the corresponding trials. The s.e.m. is shown as gray shading but is contained in the line width. **c**, Fraction of imaged cells with place fields. Place fields and properties were calculated using a matched number of engaged and disengaged trials that are closest to each other in time. Two-sided Wilcoxon signed-rank test, *P* = 8.0 × 10^−7^. *n* = 32 sessions and 8 mice. **d**, Each pair of columns corresponds to one cell. The first two cells have place fields in both engaged and disengaged trials (9,837 cells total showed similar pattern); the third cell lost its place field in disengaged trials (10,792 cells showed similar pattern); and the last cell gained a place field in disengaged trials (3,254 cells showed similar pattern). First row: cell images with cell masks outlined. Second row: trial-wise activity for 20 engaged trials immediately before a streak of 20 disengaged trials. Dark blue represents 0 activity (a.u.). Top 1% values were saturated and indicated as max activity. Third row: mean activity (black traces) across periods. Red lines indicate location of a significant place field. Gray shading indicates 99% confidence bounds for shuffled data. **e**, Left: reliability of place fields (mean trial-to-trial correlation of spatially binned activity). Two-sided Wilcoxon rank-sum test, *P* = 0. Right: reliability across position. *n* = 20,629 place fields in engaged trials; 13,091 place fields in disengaged trials. **f**, Left: selectivity of place field activity. Two-sided Wilcoxon rank-sum test, *P* = 9.6 × 10^−200^. Right: selectivity across position. *n* = 20,629 place fields in engaged trials; 13,091 place fields in disengaged trials. **g**, Representative trial-wise activity and place field selectivity of four cells around disengaged streak onsets. Dark blue represents 0 activity (a.u.), and the max activity after saturating the top 1% of values is indicated. Green vertical line marks the first disengaged trial. **h**, Selectivity change around disengaged streak onsets for cells with place fields in engaged trials. Left: shading represents mean ± s.e.m. across cells. Right: shading represents mean ± s.e.m. across sessions. *n* = 12 sessions and 6 mice. **i**, Trial-wise decoding error around disengaged streak onsets. The decoder was trained on ten mostly engaged trials and tested on 20 mostly engaged trials immediately before a disengaged streak of at least ten trials. Chance-level error is 50 cm. Left: shading represents mean ± s.e.m. across frames of each trial. Right: shading represents mean ± s.e.m. across sessions. *n* = 15 sessions and 6 mice. a.u., arbitrary unit; NS, not significant.

**Extended Data Fig. 4:**
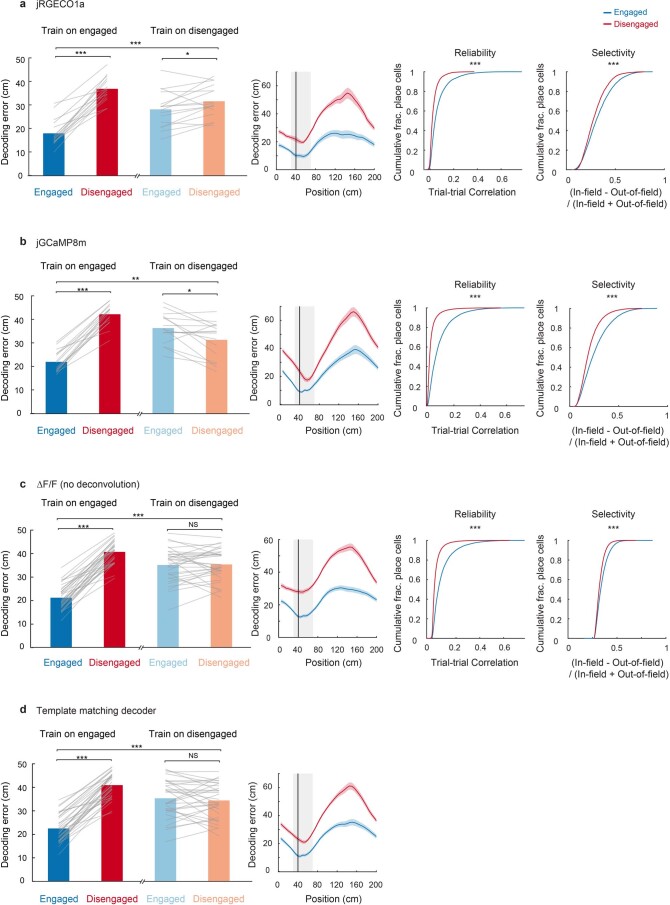
Decoding and place field quality for different calcium indicators, fluorescence trace processing, and decoding methods. All panels are plotted as in Fig. 2h-i and Fig. 3e-f. **a**. jRGECO1a sessions. Two-sided Wilcoxon signed rank test: Engaged vs. disengaged for decoder trained on engaged trials, p = 4.4 × 10^−4^. Engaged vs. disengaged for decoder trained on disengaged trials, p = 0.03. Train/test on engaged vs. train/test on disengaged, p = 4.4 × 10^−4^. Two-sided Wilcoxon rank sum test: reliability p = 0, selectivity p = 9.64 × 10^−200^. n = 16 sessions, 4 mice. Shading represents mean ± SEM. **b**. jGCaMP8m sessions. Two-sided Wilcoxon signed rank test: Engaged vs. disengaged for decoder trained on engaged trials, p = 4.4 × 10^−4^. Engaged vs. disengaged for decoder trained on disengaged trials, p = 0.02. Train/test on engaged vs. train/test on disengaged, p = 2.3 × 10^−3^. Two-sided Wilcoxon rank sum test: reliability p = 0, selectivity p = 9.64 × 10^−200^. n = 16 sessions, 4 mice. Shading represents mean ± SEM. **c**. ΔF/F instead of deconvolved activity. Two-sided Wilcoxon signed rank test: Engaged vs. disengaged for decoder trained on engaged trials, p = 8.0 × 10^−7^. Engaged vs. disengaged for decoder trained on disengaged trials, p = 0.54. Train/test on engaged vs. train/test on disengaged, p = 1.1 × 10^−6^. Two-sided Wilcoxon rank sum test: reliability p = 0, selectivity p = 5.7 × 10^−206^. n = 32 sessions, 8 mice. Shading represents mean ± SEM. **d**. Template matching decoder. Two-sided Wilcoxon signed rank test: Engaged vs. disengaged for decoder trained on engaged trials, p = 8.0 × 10^−7^. Engaged vs. disengaged for decoder trained on disengaged trials, p = 0.54. Train/test on engaged vs. train/test on disengaged, p = 1.7 × 10^−6^. n = 32 sessions, 8 mice. Shading represents mean ± SEM.

**Extended Data Fig. 5:**
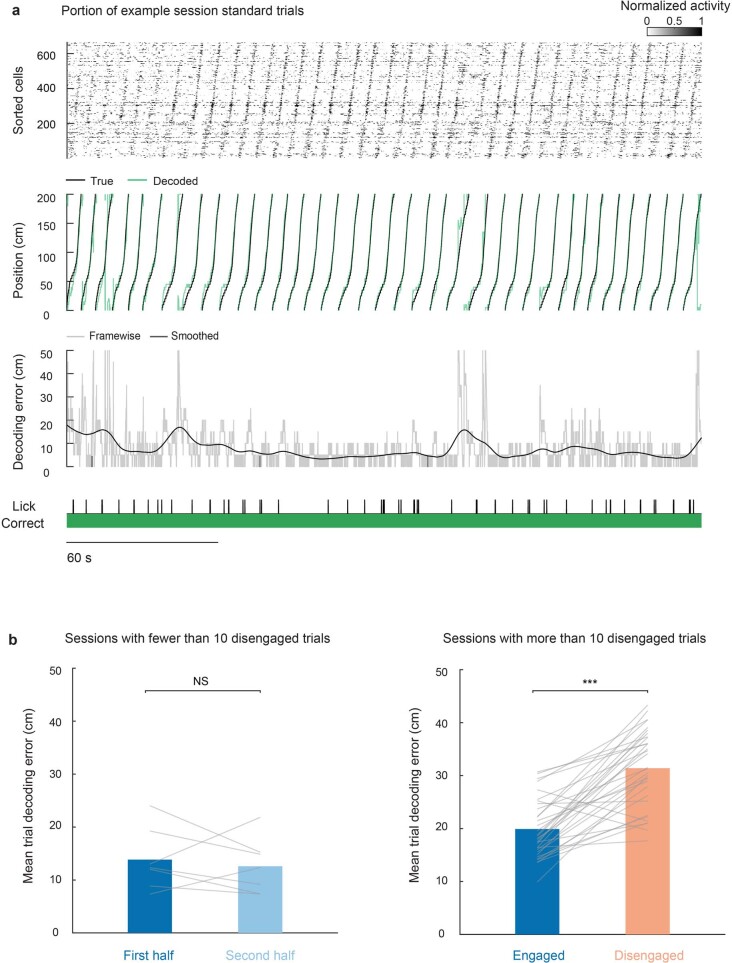
Sessions with fewer than ten disengaged trials. **a**. Portion of one example session with fewer than ten disengaged trials (standard trials only). Top: Raster plots of deconvolved activity, sorted by the location of each neuron’s maximal activity on correct trials. Only neurons whose standard deviation of activity is above the 30^th^ percentile of all neurons’ standard deviation are shown. Top 8% pixels were saturated. Decoded positions and decoding error were calculated using a sliding-window decoders as in Fig. 2f. Licks and correctness are shown at the bottom. **b**. Decoding performance of within-type held-out trials. Each gray line represents one session. Left: mean trial-wise decoding error of sessions without sufficient disengaged trials. The errors on held-out trials from the first half of the engaged trials (trained and tested on the first half) were compared to those from the second half of the engaged trials (trained and tested on the second half). Two-sided Wilcoxon signed rank test, p = 0.81. n = 7 sessions, 4 mice. Right: mean trial-wise decoding error of sessions with more than ten disengaged trials, as in Fig. 2h first and last bar. Two-sided Wilcoxon signed rank test, p = 2.2 × 10^−6^. n = 32 sessions, 8 mice.

Hippocampal neurons formed consistent place cell sequences within the engaged trials, but the profile of population activity was markedly different in the disengaged trials (Figs. 2f,g and 3b). This surprising change in neural activity, as mice performed trials with identical visual cues and continued to traverse the same maze, raised the question of whether the spatial code had remapped or degraded. We trained separate decoders to predict the mouse’s spatial position in the maze based on activity in either the engaged or disengaged trials. The decoder trained on the engaged trials accurately predicted the mouse’s location on held-out engaged trials, indicating that a robust spatial map was present (Fig. 2h, left and 2i). This decoder’s error increased by nearly 100% when tested on disengaged trials, confirming the major change in hippocampal activity. Notably, the decoder trained on the disengaged trials performed poorly when tested on other disengaged trials and was equally poor when tested on engaged trials (Fig. 2h, right). Decoding error in the disengaged trials was lower than chance levels, and, thus, the spatial code was degraded but not absent. These effects were qualitatively similar across different calcium indicators and decoding approaches (Extended Data Fig. 4). In contrast, in sessions without behavioral disengagement, the decoding accuracy was similar throughout the session (Extended Data Fig. 5). These results reveal that the spatial code degraded when the mouse voluntarily disengaged from the task despite running continuously through the same environment. Although remapping may have contributed to the reorganization, the much worse spatial decoding in disengaged trials indicates a substantial degradation of the place code.

**Extended Data Fig. 6:**
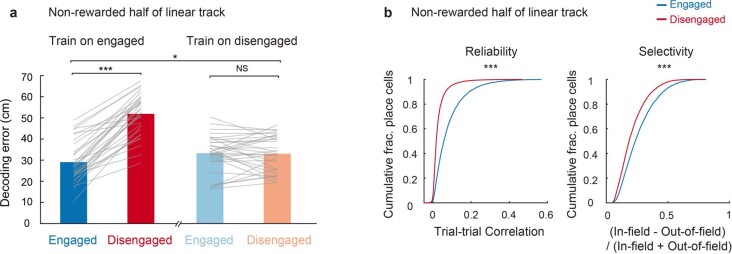
Decoding and place field characteristics for the non-rewarded half of the track. **a**. Similar to Fig. 2h, except for the non-rewarded half of the track. Two-sided Wilcoxon signed rank test: Engaged vs. disengaged for decoder trained on engaged trials, p = 8.0 × 10^−7^. Engaged vs. disengaged for decoder trained on disengaged trials, p = 0.91. Train/test on engaged vs. train/test on disengaged, p = 0.04. n = 32 sessions, 8 mice. **b**. Similar to Fig. 3e,f, except for only the non-rewarded half of the track. Two-sided Wilcoxon rank sum test: reliability p = 0, selectivity p = 1.9 × 10^−222^. n = 32 sessions, 8 mice.

**Extended Data Fig. 7:**
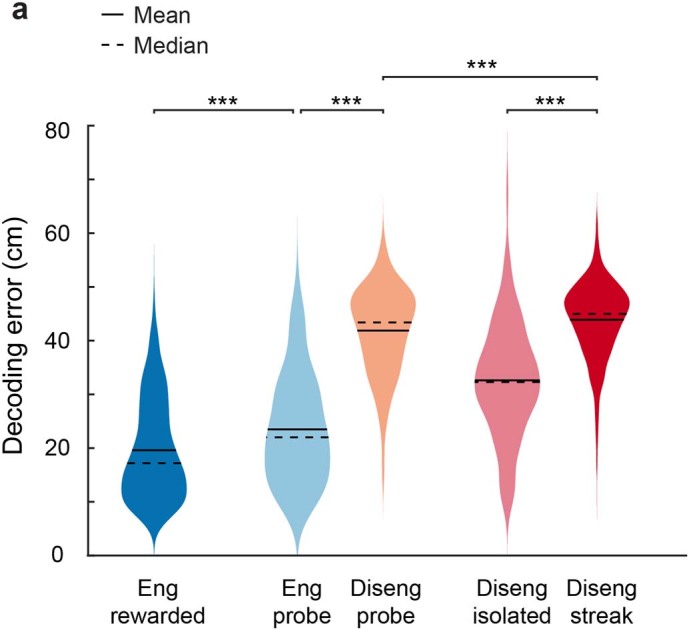
Decoding error for various trial types. **a**. Violin plots showing decoding error distributions for five trial types using the decoder from Fig. 2h trained on engaged trials. Number of trials: engaged rewarded, 3080; engaged probe, 352; disengaged probe, 429; disengaged isolated, 189; disengaged streak, 1855. Only streaks of more than ten trials were considered. Two-sided Wilcoxon rank sum test: Engaged rewarded vs. engaged probe, p = 2.1 × 10^−12^. Engaged probe vs. disengaged probe, p = 1.9 × 10^−86^. Disengaged isolated vs. disengaged streak, p = 1.3 × 10^−50^. Disengaged probe vs. disengaged streak, p = 2.3 × 10^−6^.

In the disengaged trials, mice received fewer rewards and ran at a uniform speed across the track, without slowing down as they approached the reward zone (Fig. 2j, top). If the absence of reward zone activity led to the degraded spatial code, then we expect decoding error to increase primarily near the reward zone^22^. Instead, decoding performance was worse throughout the track (Fig. 2i). Furthermore, the changes in the place code between engaged and disengaged trials were qualitatively similar when considering only the non-rewarded part of the track (Extended Data Fig. 6). We also examined decoding accuracy on probe trials, during which the reward was omitted. Although decoding error was slightly higher on probe trials compared to standard trials with correct performance, the increase in error during disengagement was over 400% higher (Extended Data Fig. 7). Therefore, peri-reward and retrospective reward effects likely cannot fully account for the change in the spatial code between engaged and disengaged trials. To test potential effects of changes in running speed, we sub-sampled from engaged and disengaged trials to match the running speeds for all spatial positions in the maze (Fig. 2j, bottom). With the running speed matched between engaged and disengaged trials, we still found a degradation of the spatial code, indicating that differences in running did not cause the change in the spatial code as the mouse disengaged from the task (Fig. 2k). Together, these results rule out two major behavioral differences between the engaged and disengaged trials and, instead, point to the mouse’s internal state as a key determinant of the spatial code.

We considered that population activity may undergo a gain change, resulting in lower activity during voluntary disengagement^10,25–27^. Instead, we found similar levels of population activity in the engaged and disengaged trials (Fig. 3a,b). Also, whereas place field tuning was highly correlated between trials within the engaged period, tuning was uncorrelated between engaged and disengaged trials (Fig. 3b). Thus, the degradation of the spatial code was more likely due to a reorganization of activity than a weakening of activity or rate remapping.

**Extended Data Fig. 8:**
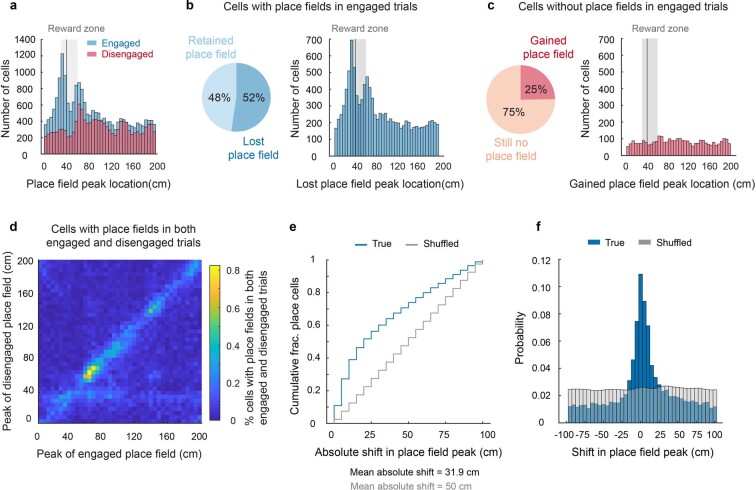
Place field peak locations and properties. **a**. Peak location of place fields in engaged and disengaged trials. n = 20,629 place fields in the engaged trials, 13,091 place fields in the disengaged trials. **b**. Left: Place field status in the disengaged trials for cells with place fields in engaged trials. n = 20,629 cells. Right: peak locations of lost place fields. **c**. Left: Place field status in the disengaged trials for cells without place fields in the engaged trials. n = 13,091 cells. Right: peak locations of gained place fields. **d**. Peak locations for cells with place fields in both engaged and disengaged trials. n = 9837 cells. **e**. Absolute shift in place field peak from engaged to disengaged trials. In the shuffled data (gray), the disengaged place field peak locations were randomly permuted 1000 times, and the absolute shifts were calculated. **f**. Histogram of true and random shifts in peak locations between engaged to disengaged trials. Same shuffling procedure as in (e).

Consistent with a reorganization of activity and degradation of the place code, despite similar levels of activity the number of place cells in the disengaged trials was approximately 35% lower (Fig. 3c). Also, 52% of cells with place fields in the engaged trials no longer had place fields when the mouse disengaged, and 25% of cells with no place field had a place field in the disengaged trials (Extended Data Fig. 8a–c). For cells with place fields in both engaged and disengaged trials, only approximately half had place fields in similar locations (Extended Data Fig. 8d–f). Although place cells were present in the disengaged trials, they had greatly reduced reliability, measured as the trial-to-trial correlation in their spatially binned activity (Fig. 3d,e). They also had less spatial selectivity, measured as the preference for activity inside the place field relative to outside the field (Fig. 3f). Therefore, as the mouse voluntarily disengaged from the task, the overall activity levels in CA1 remained similar, but the reliability and selectivity of place cells decreased, resulting in a degraded spatial code.

To understand the time course of these changes, we compared the neural activity around switches from a streak of engaged trials to a streak of disengaged trials. Some place cells underwent a marked change in activity from one trial to the next (Fig. 3g). The decoding error of the mouse’s spatial location from population activity increased within about five trials or approximately 30 seconds around the disengagement onset (Fig. 3i). Similarly, the selectivity of activity for the place field compared to locations outside the place field dropped sharply within a few trials of when the mouse behaviorally disengaged (Fig. 3g,h). Therefore, the degradation of the hippocampal code happened within a small number of trials spanning approximately less than 1 minute.

Collectively, our results reveal that the hippocampal spatial code degraded extensively when mice voluntarily disengaged from a goal-directed task. Remarkably, this degradation of the place code occurred even though the mouse experienced the identical visual cues and spatial positions as it ran continually through the virtual environment. Thus, conjunctive sensory and self-motion signals are not sufficient to form a reliable spatial map in the hippocampus, and robust place codes are not always maintained during exploration of an environment. Rather, the active engagement of an animal with its environment is essential for the maintenance of spatial maps and serves as a functional gate on the reliable firing of place cells. Our results challenge the idea that the generation of a spatial map is an automatic and unsupervised process and, instead, indicate that an animal’s internal state can profoundly affect spatial coding in the hippocampus, even in the absence of changes in the external world.

Our findings add to an emerging literature describing how internal states alter brain-wide dynamics^25,26,28,29^ by making a connection between task engagement and internal state with well-studied hippocampal place codes. Previous work has shown that place codes degrade when animals are transported on a cart or presented with open-loop replay of movement through a virtual environment^14,15,29,30^. Also, place and grid cells remap under different task demands^22,31,32^ and switch between maps of the same spatial context^33,34^. Although some of these changes may reflect differences in engagement, they are difficult to disentangle from changes in behavior and neural activity levels and may involve remapping instead of spatial code degradation. Here, we took advantage of the mouse’s voluntary disengagement from the task while it continued to run through the virtual maze, thus preserving identical sensory cues and similar movement patterns across engagement states. It is possible that the extent of disengagement we observed might be more likely in virtual reality, but even if in real-world settings the changes due to behavioral engagement are less drastic, we predict that internal states nevertheless modulate hippocampal codes.

Our results raise the possibility that an active mechanism determines the behavioral relevance of an environment and exerts top-down influence to gate spatial representations in CA1 (ref. ^35^). In this case, the change in internal state would modulate the hippocampal place code. Alternatively, the changes in place codes that we observed could arise for separate reasons and cause the change in behavior associated with disengagement. Future experiments will be needed to test the underlying mechanisms and define whether engagement is best considered as a change in attention, satiety and/or motivation. Regardless of the mechanisms, our results reveal that the hippocampus does not always maintain a spatial map and that place codes degrade even as animals experience the identical visual cues and spatial positions. This modulation of place codes could serve a general role in hippocampal function, such as to aid rapid task switching and toggling between spatial and non-spatial processing.

## Methods

### Mice

All experimental procedures were approved by the Harvard Medical School Institutional Animal Care and Use Committee and were performed in compliance with the *Guide for Animal Care and Use of Laboratory Animals*. Imaging and behavioral data were collected from four Thy1-jRGECO1a GP8.31 (ref. ^36^) (030526, Jackson Laboratory) × B6.Cg-Tg(Fos-tTA,Fos-EGFP*)1Mmay/J (018306, Jackson Laboratory) double-transgenic male mice, two B6.Cg-Tg(Fos-tTA,Fos-EGFP*)1Mmay/J transgenic male mice (018306, Jackson Laboratory) and five C57BL/6J wild-type male mice (000664, Jackson Laboratory)^36,37^. All mice were adult males at least 12 weeks old at the start of experiments. A subset of these data was collected for experiments studying Fos expression. Here, Fos expression (EGFP fluorescence) was not analyzed and will be reported in following work.

### Virtual reality and behavioral hardware

We used a miniaturized modified version of the visual virtual reality system that has been described previously^38–40^. Head-restrained mice ran on a spherical treadmill that was constrained with a yaw and roll blocker to rotate only in pitch (forward and backwards relative to the mouse’s body). Ball movement was detected by two optical sensors (ADNS-9800, Avago Technologies) connected to a Teensy 3.2 microcontroller (https://www.pjrc.com/) mounted to a custom-printed circuit board. Forward translation in the virtual environment was controlled by rotation of the ball, with velocity gain adjusted such that distance traveled in the virtual environment equaled the distance traveled on the surface of the ball. The virtual environment was back-projected (laser pico-bit projector, Celluon) onto a parabolic screen surrounding ~180° of the mouse in azimuth, with a minimum screen distance from the mouse of approximately 5 inches. Designs for the virtual reality and behavior hardware are available at https://github.com/HarveyLab/mouseVR. Water rewards were delivered via a metal spout, with a solenoid valve controlling reward timing and quantity. Licks were detected by a custom electrical circuit triggered by the mouse’s tongue. Multiple contacts made within a single iteration of the Virtual Reality Mouse Engine (ViRMEn) (~60 Hz) were considered to be a single lick event for the purposes of behavioral analysis.

### Virtual environment

Virtual environments were constructed using the ViRMEn in MATLAB^41^. Environments consisted of tracks 2 m in length. The end of the track was continuous with the beginning of the track, such that it repeated continuously in a circular topology. The walls of the track were tiled with textures to serve as visual landmarks.

### Behavior task

Before being exposed to the virtual environment, mice were habituated and trained to run and lick the water spout to receive rewards. Once transitioned into the visual environment, the task contingency was fixed, and water rewards were delivered after the first lick in the reward zone. The reward zone was 1/10th the length of the track (20 cm). Occasionally (on 0.7% of trials), manual rewards were delivered by the experimenter to ensure that lick detection and reward delivery systems were working; trials with manual rewards were excluded from further analysis. In the final version of the behavioral task, mice were required to traverse the linear track and lick in a specific reward zone to receive water rewards. Three trial types were present within each session: standard, crutch and probe. In crutch trials (0–30% of trials), a water reward was delivered as soon as the mouse entered the reward zone, regardless of licking behavior. In standard trials (60–90% of trials), a water reward was delivered after the first lick in the reward zone. In probe trials (10% of trials), no rewards were delivered, regardless of the mouse’s licking behavior. Probe trials allowed us to assess licking and running behavior in the absence of rewards. For crutch and standard trials, licks that occurred in the reward zone after the delivery of reward were deemed ‘consumption licks’ and did not contribute to measures of licking selectivity or numbers of licks. All other licks were considered non-consumption licks.

### Surgery

Before behavioral training, dental cement was used to attach a titanium head plate to the skull of a 6–8-week-old mouse, typically during the cannula implant surgery. Upon recovery, the mouse was put on a water schedule, receiving 1 ml of water in total per day. Body weight was monitored daily to ensure that it was maintained above 80% of the pre-restriction measurement.

Virus injections. Before placement on the water schedule, mice were anesthetized with isoflurane (1–2% in air) and given an injection of dexamethasone (intraperitoneal, 2 mg kg^−1^ of body weight) and buprenorphine (0.5 mg kg^−1^, ZooPharm). Three craniotomies were centered around a target 1.8 mm lateral to the midline (right hemisphere) and −2.3 mm posterior to bregma. The approximate locations of the three craniotomies were (1.55, −2.3), (1.93, −2.08) and (1.93, −2.52) mm (ML and AP axes, respectively) from bregma. Virus injections were performed using beveled glass micropipettes with tips positioned ~1.3 mm below the dura. Approximately 60 nl of AAV1/2 CAG-jRGECO1a (1 × 10^11^ titer, into B6.Cg-Tg(Fos-tTA,Fos-EGFP*)1Mmay/J mice) or pGP-AAV1-syn-jGCaMP8m-WPRE (1 × 10^12^ titer, into wild-type mice) was injected in each location. No virus injections were performed in the double-transgenic mice (Thy1-jRGECO1a x B6.Cg-Tg(Fos-tTA,Fos-EGFP*)1Mmay/J).

Cannula implant. Cannula implants for hippocampal imaging were performed on water-restricted mice at approximately 90% pre-restriction body weight. Analgesic and anesthetic procedures were carried out as described for the viral injections. The hippocampal window and head plate surgery were carried out following the procedure developed by Dombeck et al.^24,42^. An approximately 2.8-mm-diameter craniotomy was made using both a trephine drill and a hand-held dental drill, centered over the previous virus injection craniotomies. The dura was removed using a needle, micro knife and forceps (Fine Science Tools). The cortex was then aspirated slowly down to the white matter of the external capsule. During aspiration, saline was repeatedly applied to the brain. Saline irrigation was continued until all major bleeding stopped. The outer layers of the external capsule were then peeled away using light suction within the saline well, without directly touching the fibers. Irrigation with saline was continued until all bleeding stopped. A cannula was then lowered down into the craniotomy and cemented in place using Metabond dental cement. In a subset of mice, a small drop of Kwik-Sil was applied to the surface of the external capsule before the cannula was inserted. Cannulas were prepared in advance by bonding a 2.5-mm-diameter cover glass with a stainless steel tube (2.31 mm inner diameter, 2.77 mm outer diameter, 1.5 mm long) using UV-curable optical adhesive (Norland Products). During the cannula implant surgery, dental cement was used to attach a titanium head plate to the skull parallel to the surface of the hippocampal window. Upon recovery, the mouse was put back on a water schedule.

### Two-photon imaging

Data were collected using a custom-built resonant-scanning two-photon microscope. The spherical treadmill was mounted on a three-axis translation stage (Dover Motion) to position the mouse with respect to the objective. Two-photon excitation of jRGECO1a^36,43^ was achieved using a mode-locked diode-pumped femtosecond laser at 1,040 nm (YBIX, Time-Bandwidth) or 1,070 nm (Fidelity-2, Coherent). A titanium sapphire laser was used for two-photon excitation of jGCaMP8m^44^ at 920 nm (Coherent Chameleon Vision). Emitted light was filtered and collected by a GaAsP photomultiplier tube. The microscope was controlled by ScanImage 2019 (Vidrio Technologies). Images were acquired at 30 Hz at a resolution of 512 × 512 pixels corresponding to a field of view of 448 × 448 μm or 768 × 768 μm. To synchronize imaging and behavioral data, the imaging frame clock and a subset of behavioral signals were recorded in pClamp (Molecular Devices) at 1,000 Hz. After recording, behavioral signals collected in ViRMEn were synchronized with the imaging clock and downsampled to the imaging frame rate (30 Hz), using linear or nearest-neighbor interpolation.

### Maintaining the same field of view within an imaging session

Mice were head-fixed using a custom head plate holder designed for reproducible day-to-day mounting of the mouse on the ball. Once the mouse was head-fixed, the cannula and window were cleaned using multiple cycles of filtered water and light vacuum suction to remove fine dust and debris. The imaging well and cannula were filled with filtered, freshly boiled (and cooled) water to mitigate the formation of air bubbles in the cannula during imaging. The mouse was positioned under the objective, and the field of view was manually aligned with a reference image taken on day 1 of the experiment. During the imaging session, small manual adjustments were made to counter lateral and axial drift. Post hoc assessment of drift and image quality was performed by manually examining sped-up and downsampled movies of the entire experiment after motion correction. Insufficiently stable experiments were excluded before analysis of the data. Correlations for field-of-view images at different periods of the recording session were calculated as a metric for imaging stability (Extended Data Fig. 2).

### Pre-processing of imaging data and source extraction

Before source extraction, in-plane motion was corrected using a hierarchical non-rigid registration approach (https://github.com/HarveyLab/Acquisition2P_class/)^45,46^. Spatial footprints and activity traces of putative neuron sources were identified and extracted from registered movies using Suite2p (https://github.com/MouseLand/suite2p)^47^. The resulting sources were classified into two groups: putative cell body and non-cell body sources. Classification was performed using a simple convolutional neural network trained in MATLAB on manually labelled CA1 data as described previously^46^ with the exception of two output classes rather than three.

### Fluorescence trace pre-processing

Raw traces extracted by Suite2p were further processed as follows. First, fluorescence fluctuations in the surrounding neuropil of each cell were subtracted from the raw fluorescence traces (coefficient 0.8)^48^. Next, baseline fluorescence estimate was computed as the 30th percentile in a 60-second moving window. Δ*F*/*F* was computed by subtracting and dividing the raw trace by the baseline. Zero-baseline Δ*F*/*F* traces were deconvolved using OASIS (https://github.com/zhoupc/OASIS_matlab) and then smoothed with a Gaussian kernel (0.5-second standard deviation)^49^.

### Data inclusion criteria

In total, 103 sessions were recorded from 11 mice. Thirty-nine sessions from nine mice met the inclusion criteria for enough trials (at least 50 trials), good behavior performance (mean lick selectivity of all trials with licks exceeding 0.7; see ‘Behavioral analysis’ section for lick selectivity calculation) and good imaging quality and neural activity (at least 20 trials with decoding error less than 10 cm when trained on a sliding Bayesian decoder; see ‘Decoders’ section). We also required that sessions have more than ten engaged and ten disengaged trials (see ‘Behavioral analysis’ section for engagement classification) because we aimed to compare neural activity across engagement states and needed more than ten trials for our decoding analyses. In total, 32 sessions from eight mice (four with jRGECO1a and four with jGCaMP8m) met all these criteria. Within each session, we excluded trials whose duration was less than 3 seconds or more than 60 seconds (0.05% of all trials) and crutch trials with licks only in the reward zone (3.4% of all trials).

### Behavioral analysis

Analyses were performed using custom MATLAB code. We used two lick-based metrics to define task engagement. The precision of licking was calculated as lick selectivity. The total licks were approximated as the number of 5-cm spatial bins with licks, which is less sensitive to individual trials’ differences in the number of licks. For standard and crutch trials, we counted only the first lick after reward became available and excluded consumption licks. The reward zone for the lick selectivity calculations starts 10 cm before rewards became available to include anticipatory licking. We first binned licks on each trial into 2-cm-wide bins and applied Gaussian smoothing with standard deviation of 10 cm to denoise the occurrence of licks, and lick selectivity was calculated as the following:

$$lick\,selectivity = \frac{{smoothed\,licks\,in\,reward\,zone-smoothed\,licks\,in\,opposite\,zone}}{{smoothed\,licks\,in\,reward\,zone + smoothed\,licks\,in\,opposite\,zone}}$$

The lick selectivity and number of bins with licks of all trials pooled across sessions were calculated and normalized respectively before k-means clustering with two clusters. Trials in the cluster with higher lick selectivity and number of bins with licks were labeled as ‘engaged’, and trials in the other cluster were considered ‘disengaged’. Silhouette scores were computed for all trials to evaluate the quality of clustering. Among the total 8,212 trials, only five trials had negative silhouette scores, and 95% of trials had silhouette scores greater than 0.9. All sessions contained more than ten engaged trials, and 32 sessions from eight mice (four with jRGECO1a and four with jGCaMP8m) also contained more than ten disengaged trials. These 32 sessions were used in the main analyses. The remaining seven sessions that had fewer than ten disengaged trials are shown in Extended Data Fig. 5.

To illustrate that disengaged trials tended to occur in streaks, we computed the fraction of trials that fall into streaks of different length and the transition matrix between engaged and disengaged trials in Fig. 2e. The same analyses were repeated on randomly shuffled data with matching number of engaged and disengaged trials for each session, and the results were averaged over 1,000 repeats.

### Place field definition and metrics

The linear track was divided into 40 spatial bins, each 5 cm wide, for place field and decoding analyses. For each cell, we calculated the average deconvolved neural activity inside each bin and applied Gaussian smoothing with standard deviation of 10 cm. For the raster plot of each session (Figs. 1g,h and 2f), cells whose standard deviation of activity exceeded the 30th percentile of all neurons’ standard deviation were included and sorted by the location of their most active spatial bin, calculated using their activity on all correct standard trials.

Significant place cells were determined by a shuffle test. During each shuffle, the true position of the mouse was circularly shifted relative to the neural activity by a random number of ≥500 imaging frames and then divided into six chunks whose order was randomly permuted so that the activity–position relationship was perturbed while the temporal and autocorrelation structure was preserved. Neural activity was then binned by spatial positions as described above. Significant place fields consisted of at least three consecutive spatial bins (≥15 cm), and, within each bin, the true activity exceeded the 99th percentile of the shuffled activity. Only one place field was considered for each cell because very few cells (3.4%) had more than one field. Two versions of shuffling were used. The first one used only neural activity on correct standard trials and repeated shuffling 100 times to generate a qualitative comparison between the activity in the engaged and disengaged trials (Fig. 2g). The second version repeated shuffling 1,000 times and was used for more quantitative comparisons of place fields in Fig. 3c–h and Extended Data Figs. 6b and 8, where place field properties in the engaged and disengaged trials were computed separately from a matched number of trials that are closest to each other in time. Specifically, the engaged trials had the shortest distance (in number of trials) from the median disengaged trial. The peak of each place field was determined by the spatial bin with peak activity. Reliability was defined as the pairwise Pearson correlation between the activity on each trial. The in-field and out-of-field activity were computed and normalized by the number of spatial bins. Selectivity was defined as the difference between the in-field and out-of-field activity, divided by their sum. Selectivity ranged between −1 and 1 (all out-of-field firing to all in-field firing, respectively).

For all cells with place fields in engaged trials, their engaged place fields were used as masks to define in-field and out-of-field positions for the disengaged trials, and the trial-wise selectivity around disengagement onset is shown in Fig. 3g,h.

### Decoders

Population decoders were used to decode animal position from the CA1 activity^50–52^. Specifically, naive Bayesian decoders were used to decode position from the activity of all imaged neurons on individual frames within a session. The code was modified from the placeBayes function in the Buzsaki laboratory’s GitHub repository (https://github.com/buzsakilab/buzcode/blob/6418ba3b4307c673988bcf6ca44b15927fef5a7d/analysis/spikes/positionDecoding/placeBayes.m). The decoder assumed Poisson firing and independence between neurons and adopted a uniform prior for all spatial bins. Following conventions in the field, imaging frames with running velocity less than 5 cm s^−1^ were excluded because place cells are modulated by locomotion. We tuned the decoder of each session separately by inputting a spatially binned activity template based on the ‘training’ trials, and the decoder’s performance was evaluated on the held-out trials not used for training. Decoding error was defined as the absolute difference between the true spatial bin and the decoded bin and ranges between 0 and 20 spatial bins (or 0 cm and 100 cm) due to the circular nature of the linear track. Chance-level decoding was ten spatial bins or 50 cm. For each frame and each spatial bin:

$$P\left( {pos{{{\mathrm{|}}}}a_{all}} \right) = C\left( {\mathop {\prod }\limits_{i = 1}^N f_i\left( {pos} \right)^{a_i}} \right)e^{ - \tau \mathop {\sum}\nolimits_{i = 1}^N {f_i\left( {pos} \right)} }$$

where a_all_ is the activity of all cells; C is the normalization constant; τ is the temporal bin size of one frame (1/30 s); N is the total number of cells; for each cell, f_i_(pos) is the spatially binned activity template, and a_i_ is the activity on the frame. The position bin with highest conditional probability given activity of all neurons was selected as the decoded position.

Three versions of Bayesian decoders were used in the main analyses. The first decoder was used to calculate a baseline decoding performance as part of the imaging quality inclusion criteria. The decoder is trained on a sliding window of 20 trials at a time and tested on the immediate next trial. The sessions included in the main analyses have at least 20 trials whose decoding error was less than 10 cm. These trials can occur at any point in the session and do not need to occur in a streak. Figure 2f and Extended Data Fig. 5a show the decoded position of two example sessions using this sliding decoder.

The second decoder was used to compare the spatial code across different engagement states and trial types (Fig. 2h and Extended Data Figs. 4a–c, 6 and 7). After assigning trials into engaged and disengaged clusters based on licking behavior, we trained the decoder on ten trials of the same engagement type at a time. For each iteration of training, a decoder was fit and the error computed for each of the held-out trials. For each train/test analysis (for example, ‘train on engaged, test on disengaged’), the mean trial-wise decoding error was computed across train/test splits that met those conditions. To assess decoding performance in sessions with fewer than ten disengaged trials, the same decoder setup was used, but training trials were either from the first or the second half of the engaged trials (Extended Data Fig. 5b, left).

To control for the changes in the speed profile between the engaged and disengaged periods, we performed speed matching and re-ran the same decoder setup on speed-matched frames (Fig. 2j,k). Speed matching was done between two sets of ten trials of the same or opposite engagement type at a time. We first discretized the running speed into bins of 5 cm s^−1^ increments. At every location on the track (each 2-cm spatial bin), we found the overlap in the speed distribution of the two sets of trials to be matched, which served as the target number of frames for every speed bin. We then subsampled frames from the two sets of trials without replacement so that the resulting speed profiles were matched at every spatial bin, and the total number of selected frames was also matched. Each trial after speed matching now consisted of a subset of its original imaging frames. Spatially binned activity was then calculated from the speed-matched frames. One set of ten trials was used to train the decoder at a time, and the decoder was tested on its speed-matched partner trials. The mean trial-wise decoding error was again computed. Pairs of blocks with more than ten empty spatial bins (due to non-overlapping speed distributions) were excluded from the calculation of mean decoding error.

The last version of decoder was used to assess trends in decoding performance around disengaged streak onsets. For this analysis, we selected disengaged streaks of at least ten trials in length and with the 30 trials preceding these streaks being at least 80% engaged. Fifteen sessions had streaks that met this criterion. For each session, the first ten trials of the preceding trial group (trials 21–30 before the streak) were used to train the decoder, and testing was done on the 20 subsequent trials and then the disengaged trials in a streak. The mean trial-wise decoding error is reported in Fig. 3i.

In addition to using Bayesian decoders, as a control analysis we also compared the spatial code in engaged and disengaged trials using a template matching decoder, which does not assume Poisson firing. This decoder considers the similarity in population vectors. At each spatial bin in the maze, we calculated the average population activity vector from a set of training trials as the template. Then, for each time point to be decoded, we compared that time point’s population activity vector to the template at all position bins by calculating their cosine similarity. We considered the spatial bin in the template whose activity vector most closely matched that of the time point to be the decoded position. The results from this decoder and the Bayesian decoder are very similar (Extended Data Fig. 4d).

### Statistics and reproducibility

No statistical method was used to predetermine sample size. Sample sizes in terms of mice, sessions and neurons are similar to other contemporary studies in the field^4,29,30^. Of 103 sessions in 11 mice, 39 met inclusion criteria based on behavioral performance, number of trials and decoding accuracy (see ‘Data inclusion criteria’ section). As all mice were subject to the same behavioral task, experimental conditions and analysis, randomization across subjects and blinding to experimental conditions were not necessary and did not take place during the experiments or data analysis. The study originally consisted of data from six mice (jRGECO1a calcium indicator). During peer review, data were collected from an additional five mice using the more sensitive jGCaMP8m calcium indicator in C57BL/6J wild-type mice (see ‘Mice’ section). These new data were subjected to the same analyses as the data presented in the original submission. Results were successfully replicated, appearing highly consistent across the two datasets collected approximately 2 years apart in different cohorts of mice. Data are pooled across these two sets of experiments, and, for key analyses, they are shown separately in Extended Data Fig. 4. For hypothesis testing, we chose the non-parametric two-sided Wilcoxon rank-sum test and Wilcoxon signed-rank tests to avoid making normality assumptions about the data distribution. Individual data points are shown when possible.

### Reporting Summary

Further information on research design is available in the Nature Research Reporting Summary linked to this article.

## Online content

Any methods, additional references, Nature Research reporting summaries, source data, extended data, supplementary information, acknowledgements, peer review information; details of author contributions and competing interests; and statements of data and code availability are available at 10.1038/s41593-022-01050-4.

## Supplementary information

Reporting Summary

## Data Availability

Data are available on Dryad at 10.5061/dryad.2280gb5tx.

## Code availability

The code used to analyze the data is available upon reasonable request.
