# Supplementary material for: Hippocampal place codes are gated by behavioral engagement
Source: Nat Neurosci. 2022 Apr 21;25(5):561–6. doi: 10.1038/s41593-022-01050-4 (PMC9076532; doi:10.1038/s41593-022-01050-4)
Supplement: Supplementary file 1 — Reporting Summary [file 41593_2022_1050_MOESM1_ESM.pdf]

nature portfolio

Corresponding author(s): Christopher D. Harvey

Last updated by author(s): Feb 26, 2022

Reporting Summary

Nature Portfolio wishes to improve the reproducibility of the work that we publish. This form provides structure for consistency and transparency in reporting. For further information on Nature Portfolio policies, see our Editorial Policies and the Editorial Policy Checklist.

Statistics

For all statistical analyses, confirm that the following items are present in the figure legend, table legend, main text, or Methods section.

n/a	Confirmed	
☐	☒	The exact sample size (*n*) for each experimental group/condition, given as a discrete number and unit of measurement
☐	☒	A statement on whether measurements were taken from distinct samples or whether the same sample was measured repeatedly
☐	☒	The statistical test(s) used AND whether they are one- or two-sided<br>*Only common tests should be described solely by name; describe more complex techniques in the Methods section.*
☐	☒	A description of all covariates tested
☐	☒	A description of any assumptions or corrections, such as tests of normality and adjustment for multiple comparisons
☐	☒	A full description of the statistical parameters including central tendency (e.g. means) or other basic estimates (e.g. regression coefficient) AND variation (e.g. standard deviation) or associated estimates of uncertainty (e.g. confidence intervals)
☐	☒	For null hypothesis testing, the test statistic (e.g. *F*, *t*, *r*) with confidence intervals, effect sizes, degrees of freedom and *P* value noted<br>*Give P values as exact values whenever suitable.*
☐	☒	For Bayesian analysis, information on the choice of priors and Markov chain Monte Carlo settings
☒	☐	For hierarchical and complex designs, identification of the appropriate level for tests and full reporting of outcomes
☐	☒	Estimates of effect sizes (e.g. Cohen's *d*, Pearson's *r*), indicating how they were calculated

Our web collection on statistics for biologists contains articles on many of the points above.

Software and code

Policy information about availability of computer code

Data collection	Virtual reality experiments were run using VirMEn (2016-02-12; Princeton) in combination with custom code in MATLAB (2019a and 2021b; MathWorks) for behavioral data collection. Micro-controller code used for running the virtual reality rig is available here: https://github.com/HarveyLab/mouseVR. Imaging experiments utilized ScanImage (version 2019a; Vidrio Technologies) and pClamp (Molecular Devices).
Data analysis	Preprocessing of calcium movies was carried out using custom MATLAB code (https://github.com/HarveyLab/Acquisition2P_class) and Suite2P (https://github.com/MouseLand/suite2p, versions 0.6.16 and 0.10.2). All other analyses were performed in MATLAB (2019a and 2021b; MathWorks). Code for Bayesian decoding of position was adapted from https://github.com/buzsakilab/buzcode/blob/master/analysis/spikes/positionDecoding/placeBayes.m.

For manuscripts utilizing custom algorithms or software that are central to the research but not yet described in published literature, software must be made available to editors and reviewers. We strongly encourage code deposition in a community repository (e.g. GitHub). See the Nature Portfolio guidelines for submitting code & software for further information.

Data

Policy information about availability of data

All manuscripts must include a data availability statement. This statement should provide the following information, where applicable:

- Accession codes, unique identifiers, or web links for publicly available datasets
- A description of any restrictions on data availability
- For clinical datasets or third party data, please ensure that the statement adheres to our policy

The data that support the findings of this study are available on Dryad at: https://doi.org/10.5061/dryad.2280gb5tx

Field-specific reporting

Please select the one below that is the best fit for your research. If you are not sure, read the appropriate sections before making your selection.

☒ Life sciences ☐ Behavioural & social sciences ☐ Ecological, evolutionary & environmental sciences

For a reference copy of the document with all sections, see nature.com/documents/nr-reporting-summary-flat.pdf

Life sciences study design

All studies must disclose on these points even when the disclosure is negative.

Sample size	No statistical methods were used to predetermine sample sizes. The original submission contained data from 6 mice. During review, data from an additional 5 mice were collected using a different calcium indicator. The number of mice and imaging sessions were chosen to approximately match the original dataset in size. Sample sizes in terms of mice and neurons are similar to other contemporary studies in the field (for example, see references 4, 7, 8, 23, and 29).
Data exclusions	A small number of sessions were excluded due to instability in imaging quality as described in Methods. All other experiments were analyzed, with inclusion criteria for specific analyses as described in Methods.
Replication	All jRGECO1a data were collected prior to analysis. During review, experiments were replicated using the more sensitive indicator jGCaMP8m. These data subjected to the same analyses as in the original submission and results were successfully replicated. Data are pooled across these two sets of experiments, but for key analyses are shown independently in Extended Data Figure 4.
Randomization	No randomization was carried out in the experimental design, all mice were subjected to the identical behavioral paradigm and imaging setup.
Blinding	All subjects took part in the same behavioral task and experimental conditions, therefore blinding during experiments was not necessary. All analyses took place after initial manual screening of the data (i.e. for imaging quality and stability). Besides screening of imaging quality, there were no manual steps in the analyses or experiments that required blinding.

Reporting for specific materials, systems and methods

We require information from authors about some types of materials, experimental systems and methods used in many studies. Here, indicate whether each material, system or method listed is relevant to your study. If you are not sure if a list item applies to your research, read the appropriate section before selecting a response.

Materials & experimental systems

n/a	Involved in the study	
☒	☐	Antibodies
☒	☐	Eukaryotic cell lines
☒	☐	Palaeontology and archaeology
☐	☒	Animals and other organisms
☒	☐	Human research participants
☒	☐	Clinical data
☒	☐	Dual use research of concern

Methods

n/a	Involved in the study	
☒	☐	ChIP-seq
☒	☐	Flow cytometry
☒	☐	MRI-based neuroimaging

Animals and other organisms

Policy information about studies involving animals; ARRIVE guidelines recommended for reporting animal research

Laboratory animals	Imaging and behavioral data were collected from four Thy1-jRGECO1a GP8.31 (Stock No. 030526, Jackson Laboratory) x B6.Cg-Tg(Fos-tTA,Fos-EGFP*)1Mmay/J (Stock No. 018306, Jackson Laboratory) double transgenic male mice, two B6.Cg-Tg(Fos-tTA,Fos-EGFP*)1Mmay/J transgenic male mice (Stock No. 018306, Jackson Laboratory), and five C57BL/6J wild-type male mice (Stock No. 000664, Jackson Laboratory). All mice were adult male mice at least 12 weeks old at the start of experiments. Mice were housed in a 12 h: 12 h reverse light:dark cycle at an ambient temperature of 22 °C and ambient relative humidity of 50%.
Wild animals	No wild animals were used in this study.
Field-collected samples	No field-collected samples were used in this study.
Ethics oversight	All experimental procedures were approved by the Harvard Medical School Institutional Animal Care and Use Committee and were performed in compliance with the Guide for Animal Care and Use of Laboratory Animals.

Note that full information on the approval of the study protocol must also be provided in the manuscript.
